# Sheep skeletal muscle transcriptome analysis reveals muscle growth regulatory lncRNAs

**DOI:** 10.7717/peerj.4619

**Published:** 2018-04-11

**Authors:** Tianle Chao, Zhibin Ji, Lei Hou, Jin Wang, Chunlan Zhang, Guizhi Wang, Jianmin Wang

**Affiliations:** Shandong Provincial Key Laboratory of Animal Biotechnology and Disease Control and Prevention, Shandong Agricultural University, Taian, Shandong, China

**Keywords:** Transcriptome analysis, Mutton sheep, LncRNA, Skeletal muscle

## Abstract

As widely distributed domestic animals, sheep are an important species and the source of mutton. In this study, we aimed to evaluate the regulatory lncRNAs associated with muscle growth and development between high production mutton sheep (Dorper sheep and Qianhua Mutton Merino sheep) and low production mutton sheep (Small-tailed Han sheep). In total, 39 lncRNAs were found to be differentially expressed. Using co-expression analysis and functional annotation, 1,206 co-expression interactions were found between 32 lncRNAs and 369 genes, and 29 of these lncRNAs were found to be associated with muscle development, metabolism, cell proliferation and apoptosis. lncRNA–mRNA interactions revealed 6 lncRNAs as hub lncRNAs. Moreover, three lncRNAs and their associated co-expressed genes were demonstrated by cis-regulatory gene analyses, and we also found a potential regulatory relationship between the pseudogene lncRNA LOC101121401 and its parent gene FTH1. This study provides a genome-wide resolution of lncRNA and mRNA regulation in muscles from mutton sheep.

## Introduction

As one of the most important domestic animals worldwide, sheep (*Ovis aries*) are raised mainly for meat and other agricultural products. To improve the meat production of existing mutton sheep breeds, research focused on the molecular mechanisms underlying sheep skeletal muscle development is of vital interest. To identify genes that affect muscle growth rates in mutton sheep, several studies on sheep skeletal muscle growth have been performed using transcriptome sequencing (Zhang et al., 2014; Sun et al., 2016a; Sun et al., 2016b; Bidwell et al., 2014). Many regulatory genes involved in sheep muscle growth and development have already been detected and reported. However, very few of these studies have focused on long noncoding RNAs (lncRNAs) in their transcriptome analyses.

In human and mouse, lncRNAs have already been reported as important regulatory factors of muscle growth and differentiation in multiple studies (Wang et al., 2015; Mueller et al., 2015; Han et al., 2015; Ballarino et al., 2015; Legnini et al., 2014). In goat and bovine, which are both closely related to sheep, lncRNAs have also been found to have crucial functions in skeletal muscle development (Zhan et al., 2016; Jin et al., 2017; Xu et al., 2017). Discovering differentially expressed lncRNAs in skeletal muscle from different sheep breeds would enable us to have a better understanding of the regulatory functions of lncRNAs in sheep muscle growth. Obviously, identification of relevant lncRNAs would improve our understanding of the regulatory mechanisms involved in sheep muscle growth and meat production. Recently, several studies have reported the detection and analysis of lncRNAs from multiple tissues in sheep (Bidwell et al., 2014; Yue et al., 2016; Bakhtiarizadeh et al., 2016), though similar studies on sheep skeletal muscle are still needed. In our previous report, we detected lncRNA transcripts expressed in sheep skeletal muscle (Chao et al., 2016). However, in the absence of samples and reference genome annotation, the biological functions of the lncRNAs remain unknown.

In this study, we used publicly available RNA-seq data from four different sheep skeletal muscle sequencing projects to identify and functionally predict lncRNAs. Among the four projects, two were designed to study the transcriptome differences between high production mutton sheep (Dorper sheep and Qianhua Mutton Merino sheep) and low production mutton sheep (Small-tailed Han sheep), which is consistent with our research purpose and was applied when detecting differential lncRNA expression. As a cross between Black headed Persian and Dorset, Dorper is a composite breed derived from South Africa (Milne, 2000). As a world-famous mutton sheep breed, Dorper is well known for its good muscle conformation for producing a desirable carcass. The Qianhua Mutton Merino sheep is a new breed reported by Sun et al. (2016a) that showed better meat performance than its parent breeds, the South Africa Mutton Merino and Northeast Fine-wool sheep. Comparison of the above two breeds showed that Small-tailed Han sheep had lower meat production in the two corresponding studies (Zhang et al., 2014; Sun et al., 2016a). However, with extremely high litter sizes (2.61) and good meat flavour performance, Small-tailed Han sheep showed high value in mutton sheep breeding (Tu, 1989). In this study, the top priority was to search for lncRNAs that may affect muscle growth, which would provide better knowledge on the mechanisms underlying muscle development and improve meat production in low production breeds such as Small-tailed Han sheep. This study significantly advances knowledge regarding sheep skeletal muscle lncRNA expression and will also provide the basis for further mutton sheep breeding.

## Materials and Methods

### Data acquisition and filtering

No new transcriptome sequencing datasets were generated in this study. RNA-seq datasets used for differential expression analysis were downloaded from the NCBI Sequence Read Archive database (SRA) under accession numbers SRP017799 and SRP080149. Detailed animal and gender information can be found under the BioProject accessions PRJNA185414 and PRJNA335752. Moreover, 2 additional RNA-seq datasets downloaded from the European Nucleotide Archive database (ENA) under accession numbers ERP005642 and SRP031629 were used for correlation analysis. Detailed animal and gender information could be found under BioProject accessions PRJEB6169 and PRJNA223213.

Before transcriptome mapping and assembling, we performed a filtering process with the raw sequencing reads. Using Trimmomatic-0.36 (Bolger, Lohse & Usadel, 2014), adapters, leading and trailing low-quality bases, and n bases (below quality 3) were removed. Then, all the reads were scanned with a 4-base wide sliding window and cut when the average quality dropped below 15. Finally, an average quality ≥ 20 and a minimum length of 36 bp were selected for the threshold.

### Reads mapping and gene annotation

Using STAR (Dobin et al., 2013), we mapped our clean reads with the sheep reference genome Oar_v4.0 (ftp://ftp.ncbi.nlm.nih.gov/genomes/refseq/vertebrate_mammalian/Ovis_aries/all_assembly_versions/GCF_000298735.2_Oar_v4.0/GCF_000298735.2_Oar_v4.0_genomic.fna.gz). To detect the expression of the pseudogene loci, we created GTF files with genome-mapped BAM files in Cufflinks (Trapnell et al., 2010) and merged the GTF file with the sheep reference annotation GFF file (ftp://ftp.ncbi.nlm.nih.gov/genomes/refseq/vertebrate_mammalian/Ovis_aries/all_assembly_versions/GCF_000298735.2_Oar_v4.0/GCF_000298735.2_Oar_v4.0_genomic.gff.gz). All “gene_biotype=lncRNA” categories in the gene annotation GTF file were considered to be lncRNAs. Remapping, annotation and expression detection of the genes and lncRNAs were performed with STAR and RSEM (Li & Dewey, 2011).

### Transcriptome data normalization and differentially expressed genes identification

For the eight samples from PRJNA185414 and PRJNA335752, expression data normalization was performed with the R package RUVSeq (Risso et al., 2014). All genes and lncRNAs were filtered by requiring more than 2 reads in at least 5 samples. Then, we normalized the expression matrix using upper-quartile (UQ) normalization from EDASeq (Bullard et al., 2010). Finally, according to the RUVSeq-recommended parameters, 5000 in-silico empirical negative control genes were used for unwanted variation factor estimation and expression data normalization. Relative log expression analysis (RLE) and principal components analysis (PCA) were performed according to the RUVSeq manual.

Normalized gene count data were used for differential expression analysis using DESeq2 (Love, Huber & Anders, 2014). DESeq2 used the Wald test for differential expression hypothesis testing (Love, Huber & Anders, 2014). The Wald test *P*-values were then independently filtered under the null hypothesis (Bourgon, Gentleman & Huber, 2010) and adjusted for multiple testing using the procedure of Benjamini & Hochberg (1995). The significant differentially expressed genes were declared at a log2-fold change ≥0.8 and a false discovery rate (FDR) <0.05.

### Comparative sequence analysis

To identify differentially expressed lncRNAs that were already annotated in mode species (human and mouse), NCBI blast 2.7.1 was used for comparative sequence analysis. Using BLASTN, we compared our 39 lncRNAs to the Refseq RNA and non-Refseq RNA (human and mouse). With an *e*-value <0.001 as the threshold, we selected the following criteria for the comparative analysis: sequence mapping identity 75% in a covered region and 100 nt for hitting length.

### Correlation analysis for lncRNAs with protein-coding genes and functional annotation

The correlation analysis between the lncRNAs and protein-coding genes was performed with expression data from all 26 samples from the four RNA-seq projects. The Pearson correlation test was used to estimate the co-expression relationships between lncRNAs and protein-coding genes. Moreover, the *P* value of the correlation coefficient was estimated. Using the MultiExperiment Viewer (MeV) (Saeed et al., 2003), hierarchical clustering was performed with the correlation *r*-values.

Before further functional annotation with the expression data, we calculated the Pearson correlation coefficient for the differentially expressed protein-coding genes with differentially expressed lncRNAs. Co-expressed genes from three clusters were identified by applying the correlation *r*-value >0.7 and *p*-value <0.001 as the threshold. Genes that achieved the threshold with at least one lncRNA were used for GO and KEGG enrichment analysis. The GO terms and KEGG pathway enrichment was performed using The Database for Annotation, Visualization and Integrated Discovery (DAVID v 6.8, https://david.ncifcrf.gov/) (Huang, Lempicki & Sherman, 2009).

### LncRNA and protein-coding gene interaction network

We constructed an lncRNA-mRNA co-expression network with the correlation analysis results. Additionally, a Protein–protein interaction (PPI) network between the protein-coding genes was constructed based on information from STRING v.10.0 (Szklarczyk et al., 2015) and credible interactions (combined_score ≥ 0.4) were accepted for further network analysis. Using Cytoscape (version 3.5.1) (Shannon et al., 2003), the two networks were then merged as one, and the resulting network was defined as a lncRNA-gene interaction network. Interaction degree analysis was applied with Degree Sorted Layout, and all nodes were sorted with interaction degree values. Network module analysis identification was applied with the MCODE method using the MCODE plug-in in CytoScape; the node size was selected to be proportional with the interaction degree. For the MCODE analysis, the degree cut-off was selected as 2, while a node score cut-off = 0.2 and K-Core = 2 were used for Haircut Cluster finding (Bandettini et al., 2012).

### Cis-regulatory gene analysis of differentially expressed lncRNAs

The differentially expressed genes were intersected to identify the genes 50 kbp upstream or downstream of the lncRNAs. A Pearson correlation *p*-value <0.001 was selected as the threshold for cis-regulatory gene prediction.

## Results

### Normalization analysis of sheep skeletal muscle transcriptome data

In this study, skeletal muscle RNA-seq data from eight sheep (four Small-tailed Han sheep, three Qianhua Mutton Merino sheep and one Dorper sheep) were downloaded for differential expression analysis. To distinguish the samples from the different sequencing projects, we named the three Qianhua Mutton Merino sheep from PRJNA335752 as M1, M2 and M3; the three Small-tailed Han sheep from PRJNA335752 as S1, S2 and S3; the Dorper sheep from PRJNA185414 as DP; and the Small-tailed Han sheep from PRJNA185414 as SH. The downloaded raw reads were filtered using the same thresholds and the clean reads were mapped to sheep reference genome Oar 4.0 (Table S1).

**Figure 1:**
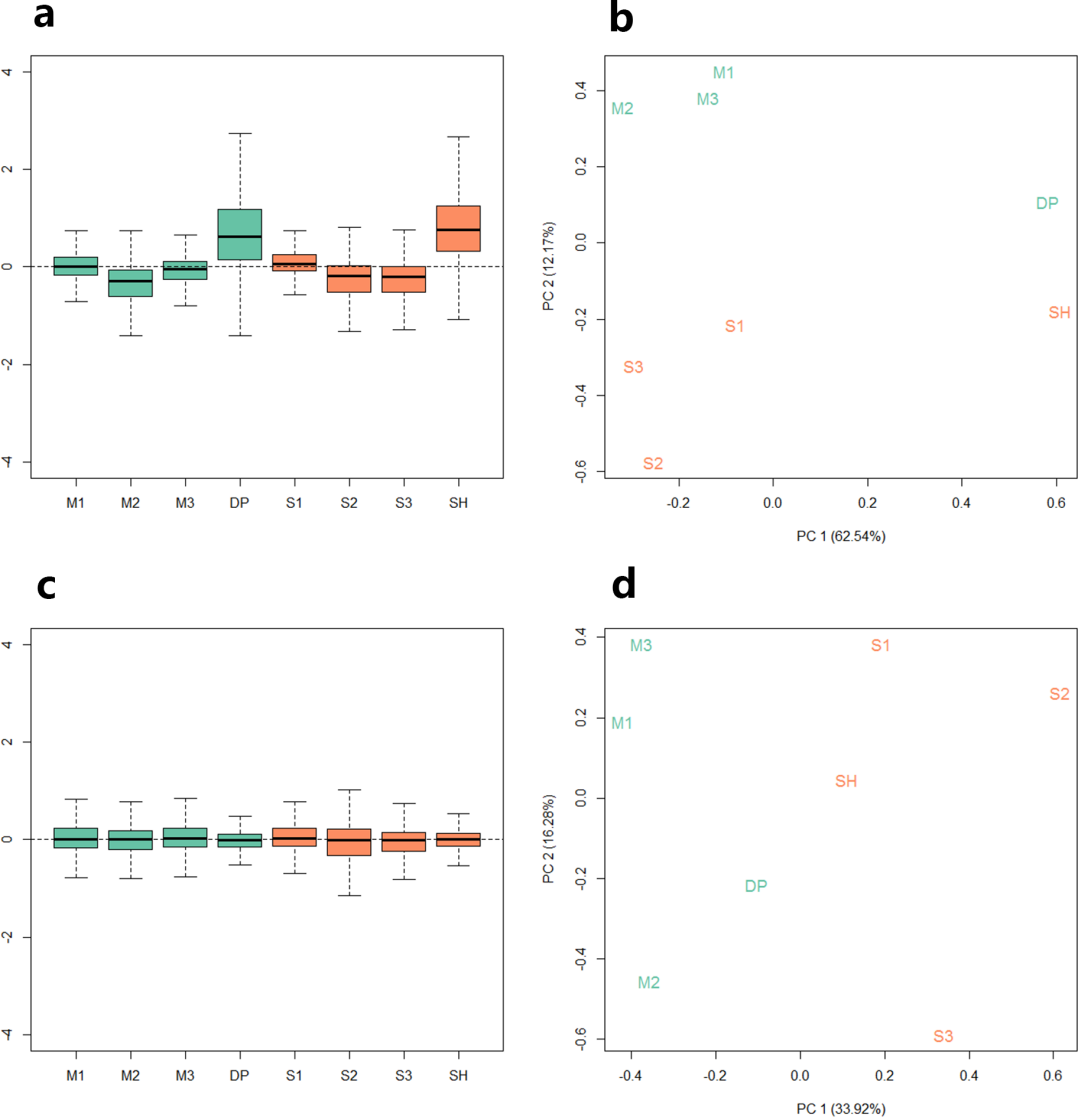
Relative log expression analysis (RLE) and principal components analysis (PCA) performed by R package RUVSeq of eight samples according to their gene counts data. Green: high production group samples. Orange: low production group samples. (A) Boxplots of un-normalized sample RLE. (B) Plots of un-normalized sample PCA. (C) Boxplots of normalized sample RLE. (D) Plots of normalized sample PCA.

Because the data we used are derived from two sequencing projects, to check the batch effect between the samples, we performed relative log expression analysis (RLE) and principal components analysis (PCA) on the eight samples according to their gene counts data. As shown in Fig. 1, the RLE boxplots (Fig. 1A) and principal component plots (Fig. 1B) reveal a clear need for between-sample normalization. Using RUVseq, the gene count data were normalized with in-silico empirical negative control genes. After normalization, the eight samples showed consistency in the relative log expression analysis (Fig. 1C). The higher production group (Mutton Merino and Dorper) and lower production group (Small-tailed Han sheep) were divided into two groups by principal component 1 (Fig. 1D).

### Identification of differentially expressed genes and lncRNA

To identify the differentially expressed genes, a comparison was performed between the higher production group (Mutton Merino and Dorper) and the lower production group (Small-tailed Han sheep). Using the DESeq2 algorithm (log2 Fold Change >0.8 and FDR <0.05), a total of 704 genes were identified as differentially expressed. In total, 386 genes were up-regulated in higher production group, while the remaining 318 genes were up-regulated in the lower production group (Table S2). Among them, 606 were known protein-coding genes, 39 were known lncRNAs, and 59 were novel gene loci. Interestingly, the top three log2-fold change value genes were all lncRNAs (LOC101101991: 5.23; LOC106991804: 4.09; and LOC105611977: −3.25).

Among the 39 differentially expressed lncRNAs, eight were detected from the pseudogene locus, while the potential features of the other 31 lncRNAs are all unknown. Performance of a comparative sequence analysis showed that only two of these 31 lncRNAs received acceptable matching results with known lncRNAs from human and mouse. LncRNA LOC105611269 showed high similarity with mouse lncRNA Nr6a1os, and, with the second highest fold change among all the differentially expressed transcripts, lncRNA LOC106991804 (log2 Fold Change = 4.09) showed high similarity with the human lncRNA LOC285847.

### Co-expression analysis between lncRNA and protein-coding genes

To reveal the genes and functions that the lncRNAs may be related to, we performed co-expression analysis between the differentially expressed lncRNAs and differentially expressed protein-coding genes. The correlation *r*-values and *p*-values are shown in Table S3.

**Figure 2:**
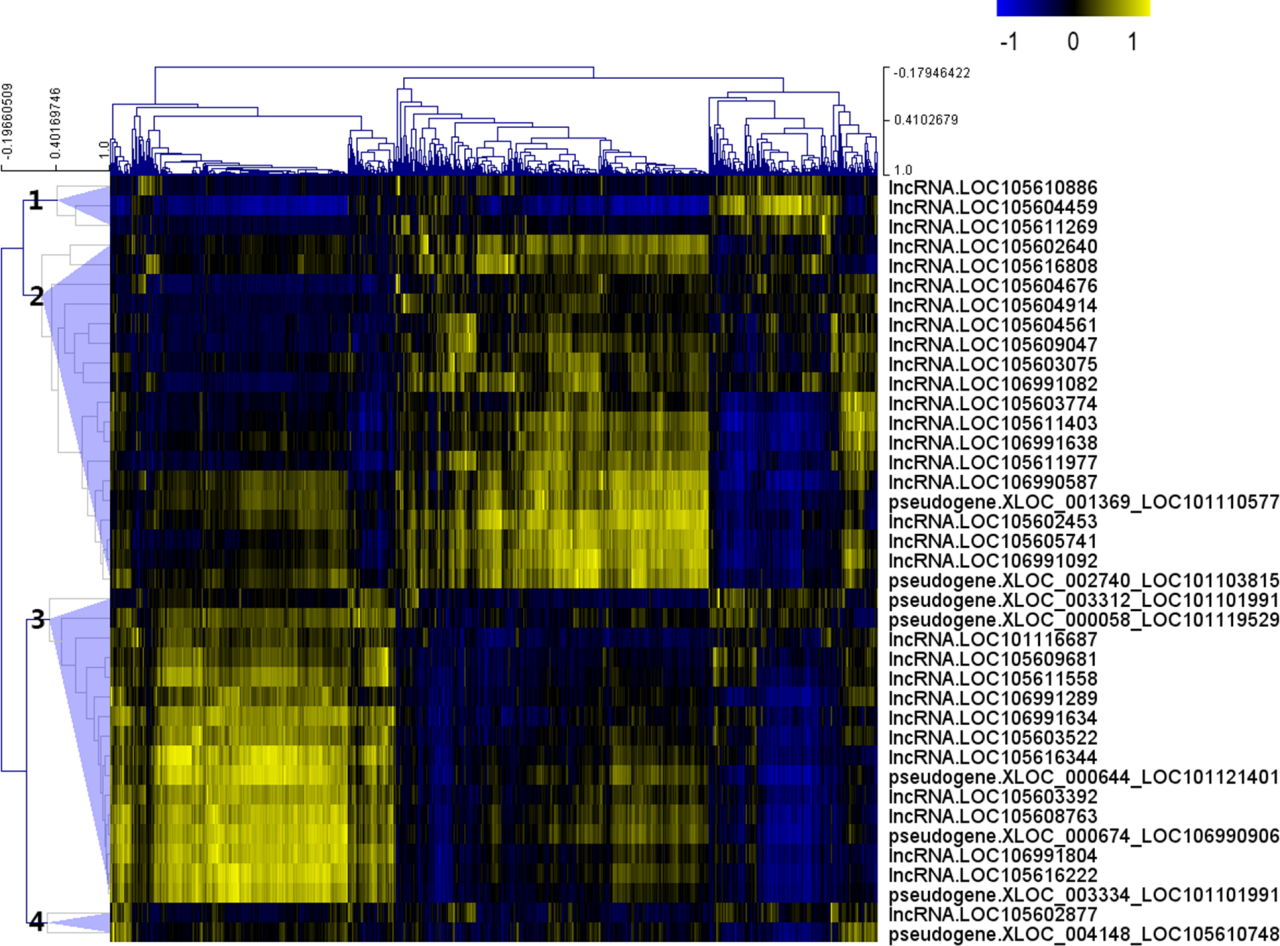
Correlation *r*-value clustering heat map perform by MultiExperiment Viewer showing the co-expression patterns of differentially expressed lncRNAs.

After the Pearson correlation analysis, hierarchical clustering was performed with the correlation *r*-values. As shown in Fig. 2, differentially expressed lncRNAs were clustered into 4 groups based on their correlation relationship with coding genes. With *r* > 0.7 and *p*-value <0.001 as the threshold, a total of 369 genes were found to be co-expressed with 32 differentially expressed lncRNAs from clusters 1 (three lncRNAs), 2 (16 lncRNAs) and 3 (13 lncRNAs), while the other seven lncRNAs (including the two lncRNAs from cluster 4) were filtered out from further analysis (Table S4).

**Figure 3:**
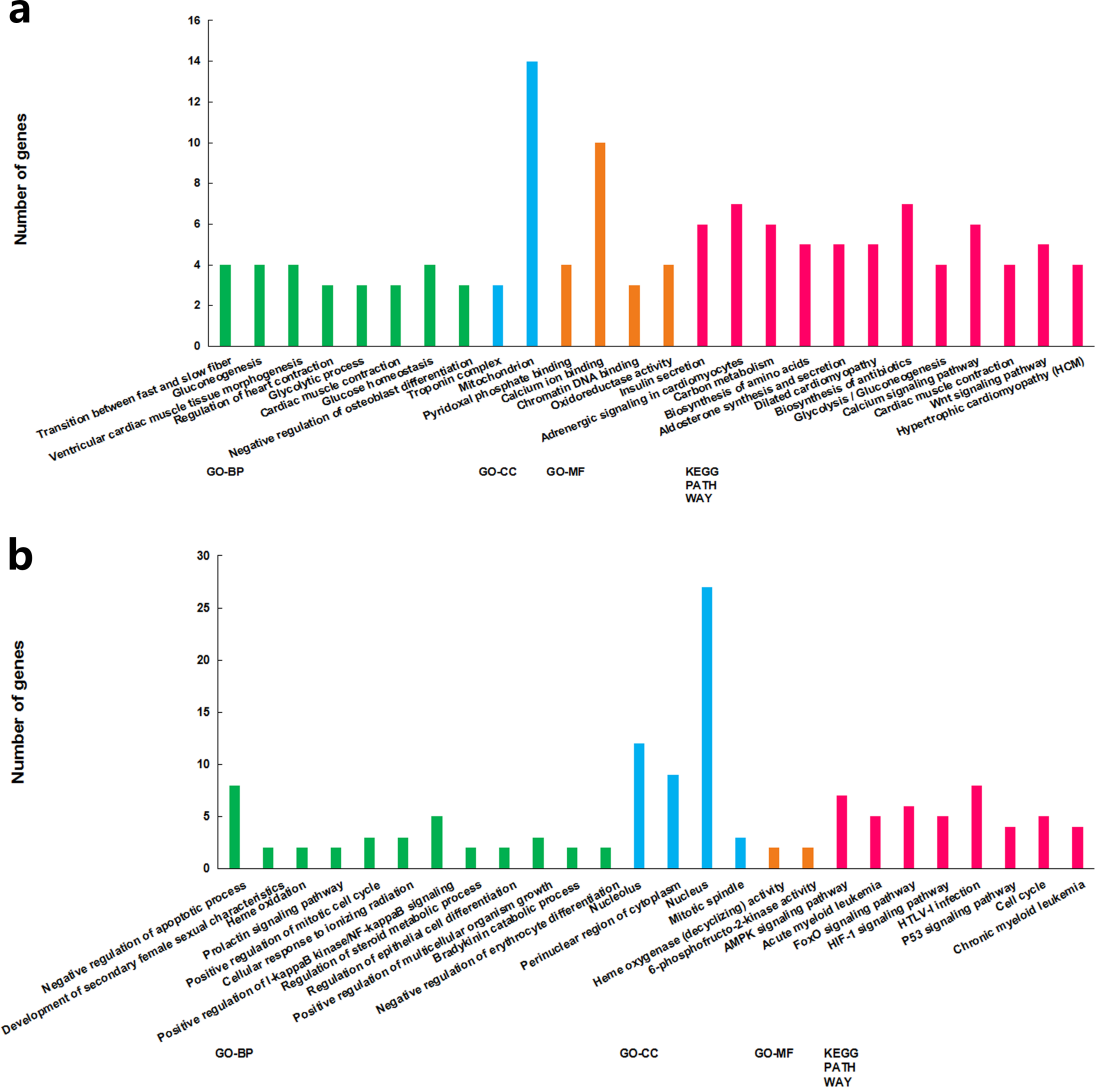
GO term and KEGG pathways achieved significant enrichment levels. Green: biological process GO term; blue: cellular component GO term. Yellow: molecular function Go term. Red: KEGG pathway. (A) Cluster 2 lncRNA co-expression genes GO term and KEGG pathway enrichment result. (B) Cluster 3 lncRNA co-expression genes GO term and KEGG pathway enrichment result.

The functions of the differentially expressed lncRNAs were predicted using GO and KEGG enrichment analyses of their co-expression genes. For cluster 1, we failed to get any significant enrichment for the co-expression genes. For cluster 2, the correlated genes were significantly enriched into 14 GO terms and 13 KEGG pathways, among which, the top enriched terms and pathways were associated with metabolism (Fig. 3A). For cluster 3, the correlated genes were significantly enriched into 19 GO terms and eight KEGG pathways, among which, the top enriched terms and pathways were mostly associated with cell proliferation and apoptosis (Fig. 3B).

### Construction of the lncRNA–gene interaction network

**Figure 4:**
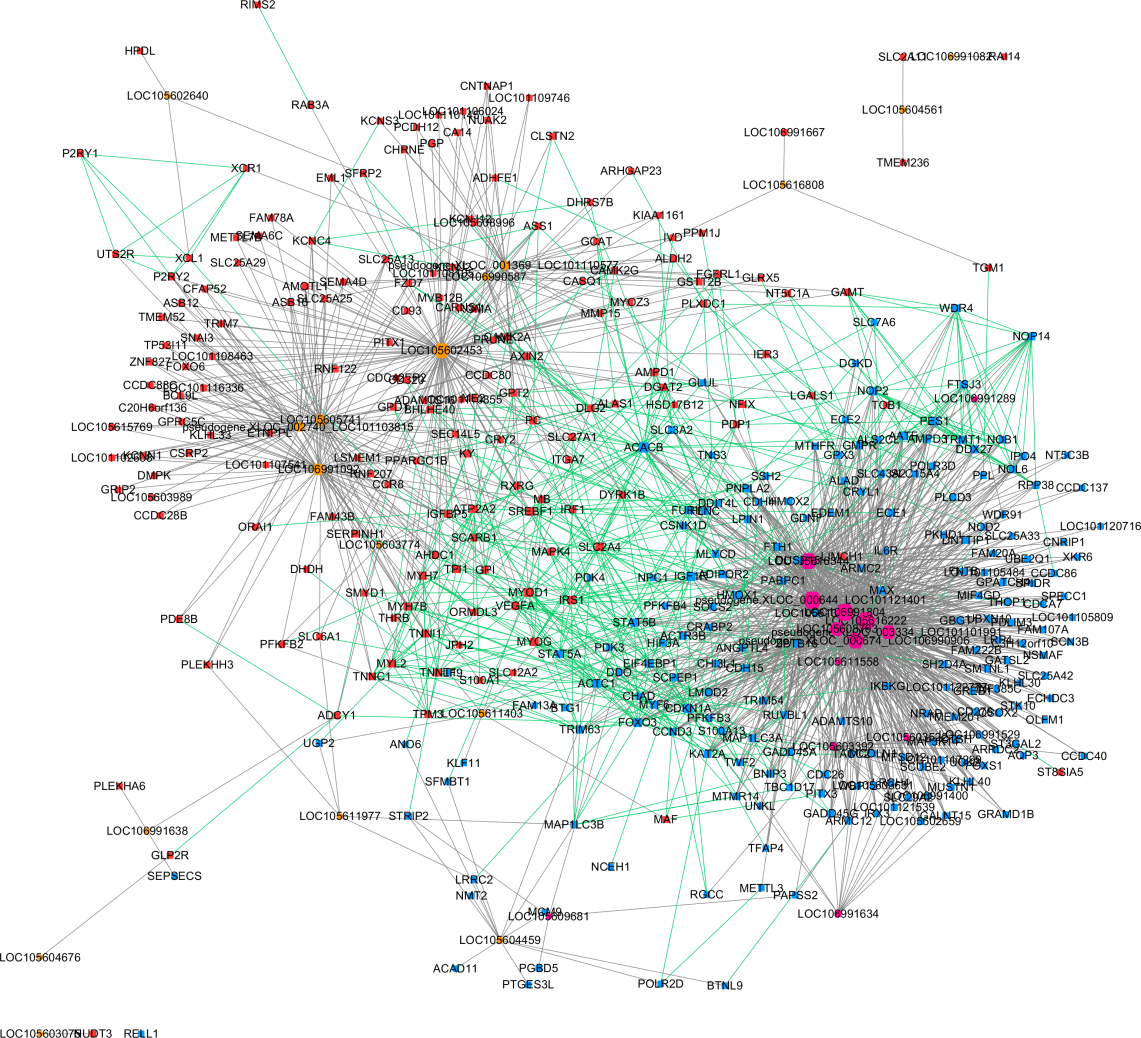
Merged lncRNA–gene interaction network created with Cytoscape. The node size was decided on the basis of the interaction degree value. Square node, Protein coding genes; Hexagon node, LncRNA nodes; Red node, Higher expressed genes in the higher production group; Blue node, higher expressed genes in the lower production group; orange node, higher expressed lncRNAs in the higher production group; purple node, higher expressed lncRNAs in the lower production group; gray edge, lncRNA-gene interaction; green edge, protein–protein interaction.

Based on the expression correlation analysis, we constructed a lncRNA-gene co-expression network with lncRNAs (from clusters 1, 2 and 3) and their correlated genes. A total of 1,206 interactions between 32 lncRNAs and 369 genes were observed. Then, we performed a Protein–protein interaction analysis with the 369 genes that were significantly correlated with lncRNAs. A total of 526 interactions between those genes were obtained from STRING. These two networks were merged as a single lncRNA–gene interaction network (Fig. 4). The merged network was constructed by 391 nodes (185 expressed higher in the higher production group and 206 expressed higher in the lower production group) and 1,726 interactions. Among the 391 nodes, 30 were lncRNAs and 361 were gene nodes. All the gene nodes and lncRNA nodes were sorted with interaction degrees (Table S5), and the top 12 interaction degree nodes are all lncRNAs, with all five pseudogene lncRNAs included.

**Figure 5:**
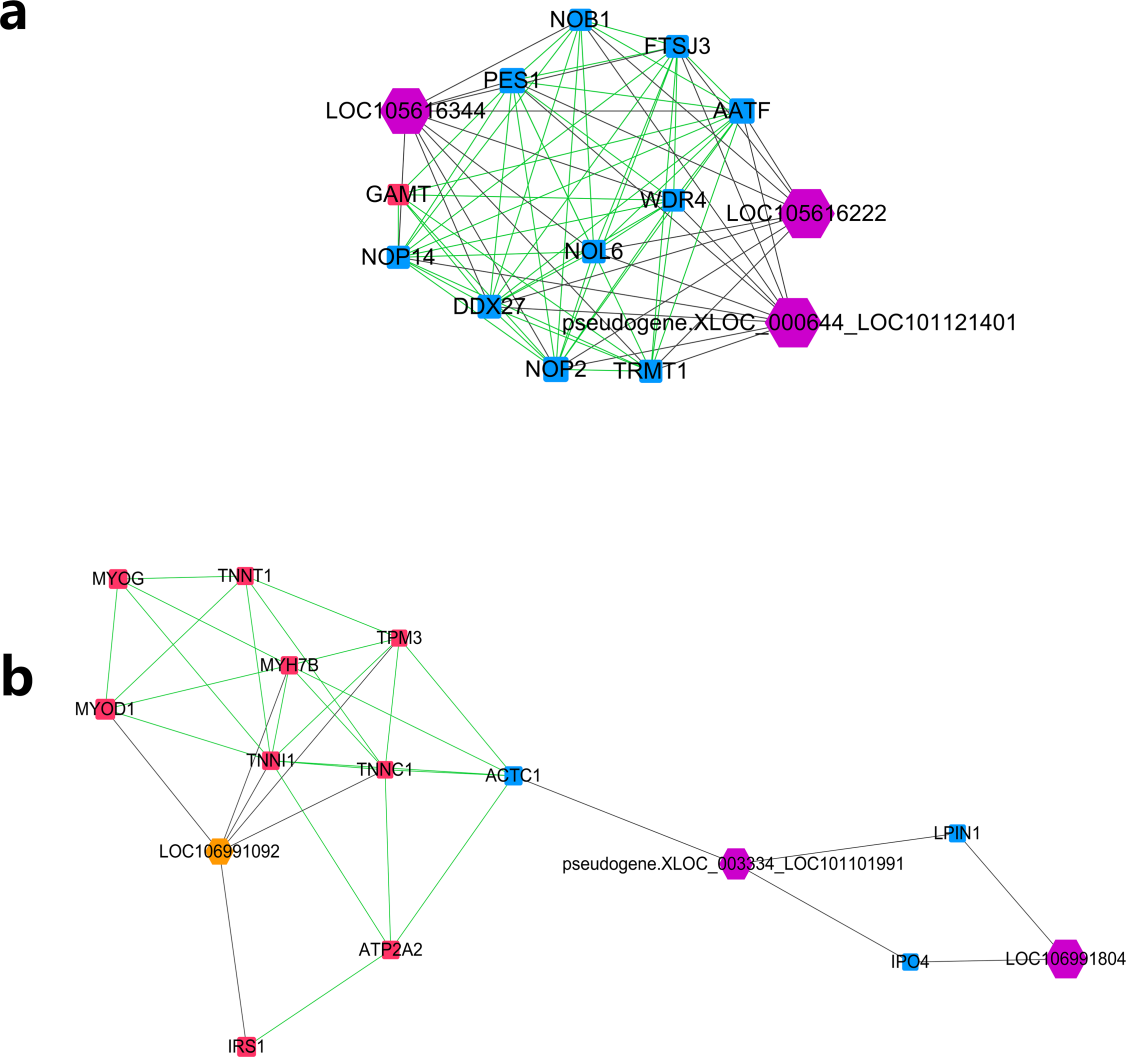
Module analysis performed by plugin MCODE of CytoScape on merged lncRNA–gene interaction network. Square node, protein coding genes; hexagon node, lncRNA nodes; red node, higher expressed genes in the higher production group; blue node, higher expressed genes in the lower production group; orange node, higher expressed lncRNAs in the higher production group; purple node, higher expressed lncRNAs in the lower production group; gray edge, lncRNA-gene interaction; green edge, protein–protein interaction. (A) LncRNA–gene interaction network Module A. (B) LncRNA–gene interaction network Module B.

Further MCODE analysis revealed that there are 8 modules in the network, among which only the top 2 modules showed scores ≥5. We named these two modules module A (Fig. 5A) and module B (Fig. 5B). In module A, all 14 nodes, except for the GAMT gene, showed higher expression in the lower production group (Small-tailed Han sheep). Protein-coding genes from module A could be significantly enriched into 2 GO terms, poly(A) RNA binding (FDR = 0.00024) and nucleolus (FDR = 0.0023). As three of the highest interaction degree nodes, lncRNAs LOC101121401 (first highest degree node), LOC105616222 (second highest degree node) and LOC105616344 (fourth highest degree node) were all included in module A. In module B, 10 nodes showed higher expression in higher production group (Mutton Merino and Dorper), and the other five nodes were expressed higher in lower production group (Small-tailed Han sheep). Protein-coding genes from module B could be significantly enriched into two GO terms, transition between fast and slow fibre (FDR = 0.0075) and troponin complex (FDR = 0.011). We also found three lncRNAs in module B, including LOC106991804 (third highest degree node), LOC101101991 (seventh highest degree node) and LOC106991092 (ninth highest degree node). As a result, these six lncRNAs were identified as hub lncRNAs.

### Regulatory analysis of differentially expressed lncRNA

To detect potential lncRNA cis-regulatory genes, we identified the chromosomal co-expression of genes 50 kbp upstream and downstream of the 32 lncRNAs. As a result, 3 cis-regulatory genes of 3 lncRNAs were detected (LOC106990587 with TRIM7, LOC105603392 with KLHL40, and LOC106991804 with ARMC12). These genes were considered potential lncRNA cis-regulatory genes. Among these three lncRNAs, only LOC106990587 showed higher expression in the higher production group (Mutton Merino and Dorper).

Of the 32 lncRNAs, five were pseudogene transcripts. Among them, we detected one pseudogene exhibiting co-expression with its parent gene. With a Pearson *r* value = 0.88, pseudogene LOC101121401 and its parent gene FTH1 were both expressed higher in the lower production group (Small-tailed Han sheep). It is worth noting that FTH1 showed the highest expression of all the differentially expressed genes, while LOC101121401 had the third highest expression among the differentially expressed lncRNAs.

## Discussion

Sheep skeletal muscle gene profiling studies have had enormous impacts on our understanding of muscle growth, providing the identification of novel regulators of skeletal muscle gene expression and function and defining the pathways that interplay to promote mutton sheep production. In this study, we performed a transcriptome level analysis of lncRNAs in skeletal muscle tissues from high production mutton sheep (Dorper sheep and Qianhua Mutton Merino sheep) and low production mutton sheep (Small-tailed Han sheep). With the goal of identifying genes affecting muscle production in mutton sheep, similar experimental designs were used by both of the studies (Zhang et al., 2014; Sun et al., 2016a) that were selected as our data source. However, because the experiments were performed with different muscles (the Longissimus, a back muscle with high commercial value, and the Biceps brachii, a shoulder muscle) and read types (single-end vs. paired-end), the statistical power of our design might be weakened, though the exact degree of weakening is still unknown. To solve this problem, a normalization analysis was performed to eliminate the existing batch effect. However, the unbalanced data design and normalization might still be a weakness of our study. Furthermore, similar problems were also present among the samples used for the co-expression analysis, where six samples were from Longissimus dorsi and biceps from three Texel sheeps (PRJEB6169) and 12 samples were from undefined muscles from crossbred sheep harbouring a callipyge mutation or not (PRJNA223213). Further verification experiments on lncRNA expression and lncRNA-gene regulatory relationships will be performed to confirm the discoveries from our in silico analysis.

Compared with the two data source studies (Zhang et al., 2014; Sun et al., 2016a), the most important innovation in this research is the detection of differentially expressed lncRNAs. A number of studies (Wang et al., 2015; Mueller et al., 2015; Han et al., 2015; Ballarino et al., 2015; Legnini et al., 2014) have proven that lncRNAs can regulate muscle growth and differentiation through cis-regulatory, trans-regulatory or competing endogenous pathways, which indicates that lncRNAs could be important muscle growth regulatory factors and potential valuable molecular marker regions for mutton sheep breeding. In our previous report (Chao et al., 2016), we performed a novel lncRNA detection analysis with sequencing data from Zhang et al. (2014), though the functions of the identified lncRNAs remain unknown. It is worth noting that the number of differentially expressed lncRNAs detected in this study is far lower than our previous report (Chao et al., 2016), which may be caused by the different methods and the statistical power. Unlike protein-coding genes, the functions of the lncRNAs could not be annotated or predicted with functional enrichment analysis. In this study, to confirm the functions of the differentially expressed lncRNAs, we applied co-expression analysis to seek potential related genes. GO and KEGG pathway enrichment revealed that the co-expression genes primarily include those known to be related to muscle development, metabolism, cell proliferation and apoptosis. These findings suggest a role for lncRNAs in the growth of sheep skeletal muscle.

Although some pseudogenes have been reported as nonfunctional, ‘dying’ genes (Zhu et al., 2007), others have been found to be transcribed as noncoding RNAs (Emadi-Baygi et al., 2017). Pseudogene RNAs can sequester microRNAs, RNA-binding proteins or translation machinery (Poliseno, 2012), as well as produce natural antisense transcripts, which fine-tune the expression of the corresponding sense transcripts at the epigenetic, transcriptional or posttranscriptional levels (Emadi-Baygi et al., 2017; Morris, 2009). In this study, we specifically examined the correlation between pseudogene lncRNA expression and their corresponding genes. Among them, only LOC101121401 was co-expressed with its corresponding gene, FTH1. A major function of FTH1 is the storage of iron in a soluble and nontoxic state (Arosio, Ingrassia & Cavadini, 2009); FTH1 was also found to be associated with several diseases (Xu et al., 2014; Chekhun et al., 2014; Li et al., 2015). Excessive iron accumulation may cause the nitration of tyrosine residues, resulting in extensive protein damage or iron-mediated nucleic acid damage, thereby leading to muscle damage (Bian et al., 2003; Xu et al., 2012). Therefore, although there is still no direct evidence, FTH1 may regulate muscle growth through its effect on iron homeostasis. Furthermore, several studies have proven that the FTH1 gene can negatively regulate cell proliferation in human (Cozzi et al., 2000; Feng et al., 2012). In this study, FTH1 showed the highest expression among all the differentially expressed genes, which is consistent with another report on the high expression of FTH1 in skeletal muscle (Polonifi et al., 2010). This gene has also been detected with multiple pseudogenes, though their functions are still unknown. Displaying a similar expression pattern, LOC101121401 may regulate the expression of FTH1 and affect muscle growth. However, since the sequence characteristics of pseudogenes are highly similar to protein-coding genes, we cannot completely rule out possibility of an incorrect mapping between them. More experiments and evidence will be required to confirm their accuracy and regulatory relationships.

For the 31 differentially expressed non-pseudogene lncRNAs, only two lncRNAs (LOC105611269 and LOC106991804) showed high similarity with human or mouse lncRNAs. However, to our knowledge, there are no reports on the functions of these lncRNAs to date. LOC285847 has been reported as a sensitivity molecular signature for proliferative diabetic retinopathy (Pan et al., 2016). In our research, LOC106991804 showed higher expression in Small-tailed Han sheep muscle and co-expressed with 124 genes, and its co-expression genes were enriched for AMPK signalling, FoxO signalling, HIF-1 signalling, cell cycle and p53 signalling pathways. It is worth noting that LOC106991804 has been detected as a hub lncRNA in network analysis, and it has also shown a potential cis-regulatory relationship with the ARMC12 gene. These results indicate that LOC106991804 could play important roles regulating apoptosis in skeletal muscle, though its high expression may be negatively correlated with muscle growth.

Further study of the merged network with MCODE revealed eight modules, among which two top score modules were selected and analysed based on GO enrichment. For the first module, module A, most protein-coding genes were significantly enriched into two RNA editing-related GO terms, while their related lncRNAs were expressed higher in Small-tailed Han sheep. In the next module, module B, only three genes (TNNT1, TNNI1 and TNNC1) were significantly enriched into two muscle contraction-related GO terms. As slow skeletal muscle troponin genes, TNNT1, TNNI1 and TNNC1 were reported with consistent expression patterns in sheep (Sun et al., 2016b); they have also been reported as important genes for maintaining slow myofibres (Pierzchala et al., 2014). These results showed that genes in module B appear to be correlated with muscle type, and their only related lncRNA in this module, LOC106991092, could be related to skeletal muscle type determination or maintenance.

An important feature of noncoding RNAs is the capacity for cis-regulation (Guttman et al., 2009). It has been reported that lncRNAs function through cis-regulation of nearby protein-coding genes as a common mechanism (Nagel et al., 2014). In the present study, three lncRNAs showed potential cis-regulation with nearby coding genes. Among them, only LOC106990587 and its cis-regulation gene TRIM7 showed higher expression in the high production mutton sheep. TRIM7 was first identified as glycogenin interacting protein (GNIP) and reported to have a high expression in skeletal muscle (Skurat et al., 2002). TRIM7 also mediates c-Jun/AP-1 activation through Ras signalling and showed ubiquitin ligase activity towards RACO-1 (Chakraborty et al., 2015). It can be inferred that the cis-regulation between LOC106990587 and TRIM7 may increase cell proliferation and promote the growth of skeletal muscle. The other two lncRNAs, LOC105603392 and LOC106991804, showed higher expression in low production mutton sheep. For LOC106991804 and its cis-regulation gene ARMC12, we could not find any relevant studies. Their differential expression may indicate that they play a role in skeletal muscle growth, though more tests are required to reveal their detailed functions. The KLHL40 gene, which is predicted to be a cis-regulation gene of LOC105603392 in this study, was reported as a muscle-specific transcript gene locus (Garg et al., 2014). A number of studies have verified that KLHL40 is a key regulatory control gene in sarcomere thin filament growth (Chen et al., 2016; Seferian et al., 2016; Winter et al., 2016), though its impact on muscle production is still unknown. Our results indicate that LOC105603392 may affect muscle growth through the cis-regulation of KLHL40.

Overall, our computational analysis revealed a number of potential muscle growth/ production-related lncRNAs in mutton sheep. These findings will provide novel insights into transcriptional level research on mutton sheep skeletal muscle systems.

## Conclusions

In summary, a total of 39 differentially expressed lncRNAs were detected in this study. Subsequent bioinformatics analyses revealed that 29 of these lncRNAs were associated with muscle development, metabolism, cell proliferation and apoptosis. Six lncRNAs were detected as hub lncRNAs, and four lncRNAs showed potential regulatory relationships with specific genes. Our research therefore provides the basis for further functional studies focused on the roles of lncRNAs in sheep skeletal muscle growth and mutton sheep production.

##  Supplemental Information

10.7717/peerj.4619/supp-1 Table S1 Statistic summary results of 26 samples from 4 sequencing projects Sequencing project and Biosample information were collected from NCBI SRA database. Data file filtering was performed with Trimmomatic-0.36.

10.7717/peerj.4619/supp-2 Table S2 Differentially expressed genes and lncRNAs Differential expression analysis was performed with DESeq2.

10.7717/peerj.4619/supp-3 Table S3 Correlation *r*-value and *p*-value between differentially expressed lncRNA and differentially expressed protein coding genes Sheet 1: Pearson correlation *r*-value matrix. Sheet 2: Pearson correlation *p*-value matrix.

10.7717/peerj.4619/supp-4 Table S4 Information of lncRNAs and co-expressed genes Differentially expressed lncRNAs from Cluster 1, 2 and 3 were contained in. Cis-regulatory genes were identified with 50 k bp upstream or downstream of the lncRNAs and a Pearson correlation *p*-value <0.001.

10.7717/peerj.4619/supp-5 Table S5 Network node interaction degree list Interaction degree and MCODE score of 391 nodes were obtained with Cytoscape 3.5.1. All nodes were ranked with interaction degree.

## Additional Information and Declarations

### Data Availability

The following information was supplied regarding data availability: All transcriptome sequencing data used in this research were introduced from other reports, and are publicly available. The database IDs of raw data are included in the manuscript (in the first paragraph of Materials and Methods).

## References

[ref-1] Arosio P, Ingrassia R, Cavadini P (2009). Ferritins: a family of molecules for iron storage, antioxidation and more. Biochimica et Biophysica Acta—General Subjects.

[ref-2] Bakhtiarizadeh MR, Hosseinpour B, Arefnezhad B, Shamabadi N, Salami SA (2016). In silico prediction of long intergenic non-coding RNAs in sheep. Genome.

[ref-3] Ballarino M, Cazzella V, D’Andrea D, Grassi L, Bisceglie L, Cipriano A, Santini T, Pinnarò C, Morlando M, Tramontano A, Bozzoni I (2015). Novel long noncoding RNAs (lncRNAs) in myogenesis: a miR-31 overlapping lncRNA transcript controls myoblast differentiation. Molecular and Cellular Biology.

[ref-4] Bandettini WP, Kellman P, Mancini C, Booker OJ, Vasu S, Leung SW, Wilson JR, Shanbhag SM, Chen MY, Arai AE (2012). MultiContrast Delayed Enhancement (MCODE) improves detection of subendocardial myocardial infarction by late gadolinium enhancement cardiovascular magnetic resonance: a clinical validation study. Journal of Cardiovascular Magnetic Resonance.

[ref-5] Benjamini Y, Hochberg Y (1995). Controlling the false discovery rate: a practical and powerful approach to multiple testing. Journal of the Royal Statistical Society B.

[ref-6] Bian K, Gao Z, Weisbrodt N, Murad F (2003). The nature of heme/iron-induced protein tyrosine nitration. Proceedings of the National Academy of Sciences of the United States of America.

[ref-7] Bidwell CA, Waddell JN, Taxis TM, Yu H, Tellam RL, Neary MK, Cockett NE (2014). New insights into polar overdominance in callipyge sheep. Animal Genetics.

[ref-8] Bolger AM, Lohse M, Usadel B (2014). Trimmomatic: a flexible trimmer for Illumina sequence data. Bioinformatics.

[ref-9] Bourgon R, Gentleman R, Huber W (2010). Independent filtering increases detection power for high-throughput experiments. Proceedings of the National Academy of Sciences of the United States of America.

[ref-10] Bullard JH, Purdom E, Hansen KD, Dudoit S (2010). Evaluation of statistical methods for normalization and differential expression in mRNA-Seq experiments. BMC Bioinformatics.

[ref-11] Chakraborty A, Diefenbacher ME, Mylona A, Kassel O, Behrens A (2015). The E3 ubiquitin ligase Trim7 mediates c-Jun/AP-1 activation by Ras signalling. Nature Communications.

[ref-12] Chao T, Wang G, Wang J, Liu Z, Ji Z, Hou L, Zhang C (2016). Identification and classification of new transcripts in dorper and small-tailed han sheep skeletal muscle transcriptomes. PLOS ONE.

[ref-13] Chekhun SV, Lukyanova NY, Shvets YV, Burlaka AP, Buchynska LG (2014). Significance of ferritin expression in formation of malignant phenotype of human breast cancer cells. Experimental Oncology.

[ref-14] Chen TH, Tian X, Kuo PL, Pan HP, Wong LJC, Jong YJ (2016). Identification of KLHL40 mutations by targeted next-generation sequencing facilitated a prenatal diagnosis in a family with three consecutive affected fetuses with fetal akinesia deformation sequence. Prenatal Diagnosis.

[ref-15] Cozzi A, Corsi B, Levi S, Santambrogio P, Albertini A, Arosio P (2000). Overexpression of wild type and mutated human ferritin H-chain in HeLa cells: *In vivo* role of ferritin ferroxidase activity. Journal of Biological Chemistry.

[ref-16] Dobin A, Davis CA, Schlesinger F, Drenkow J, Zaleski C, Jha S, Batut P, Chaisson M, Gingeras TR (2013). STAR: Ultrafast universal RNA-seq aligner. Bioinformatics.

[ref-17] Emadi-Baygi M, Sedighi R, Nourbakhsh N, Nikpour P (2017). Pseudogenes in gastric cancer pathogenesis: a review article. Briefings in Functional Genomics.

[ref-18] Feng Y, Liu Q, Zhu J, Xie F, Li L (2012). Efficiency of ferritin as an MRI Reporter gene in NPC cells is enhanced by iron supplementation. Journal of Biomedicine and Biotechnology.

[ref-19] Garg A, O’Rourke J, Long C, Doering J, Ravenscroft G, Bezprozvannaya S, Nelson BR, Beetz N, Li L, Chen S, Laing NG, Grange RW, Bassel-Duby R, Olson EN (2014). KLHL40 deficiency destabilizes thin filament proteins and promotes Nemaline myopathy. Journal of Clinical Investigation.

[ref-20] Guttman M, Amit I, Garber M, French C, Lin MF, Feldser D, Huarte M, Zuk O, Carey BW, Cassady JP, Cabili MN, Jaenisch R, Mikkelsen TS, Jacks T, Hacohen N, Bernstein BE, Kellis M, Regev A, Rinn JL, Lander ES (2009). Chromatin signature reveals over a thousand highly conserved large non-coding RNAs in mammals. Nature.

[ref-21] Han X, Yang F, Cao H, Liang Z (2015). Malat1 regulates serum response factor through miR-133 as a competing endogenous RNA in myogenesis. FASEB Journal.

[ref-22] Huang DW, Lempicki RA, Sherman BT (2009). Systematic and integrative analysis of large gene lists using DAVID bioinformatics resources. Nature Protocols.

[ref-23] Jin CF, Li Y, Ding XB, Li X, Zhang LL, Liu XF, Guo H (2017). Lnc133b, a novel, long non-coding RNA, regulates bovine skeletal muscle satellite cell proliferation and differentiation by mediating miR-133b. Gene.

[ref-24] Legnini I, Morlando M, Mangiavacchi A, Fatica A, Bozzoni I (2014). A feedforward regulatory loop between HuR and the long noncoding RNA linc-MD1 controls early phases of myogenesis. Molecular Cell.

[ref-25] Li B, Dewey CN (2011). RSEM: accurate transcript quantification from RNA-Seq data with or without a reference genome. BMC bioinformatics.

[ref-26] Li W, Garringer HJ, Goodwin CB, Richine B, Acton A, VanDuyn N, Muhoberac BB, Irimia-Dominguez J, Chan RJ, Peacock M, Nass R, Ghetti B, Vidal R (2015). Systemic and cerebral iron homeostasis in ferritin knock-out Mice. PLOS ONE.

[ref-27] Love MI, Huber W, Anders S (2014). Moderated estimation of fold change and dispersion for RNA-seq data with DESeq2. Genome Biology.

[ref-28] Milne C (2000). The history of the Dorper sheep. Small Ruminant Research.

[ref-29] Morris KV (2009). Long antisense non-coding RNAs function to direct epigenetic complexes that regulate transcription in human cells. Epigenetics.

[ref-30] Mueller AC, Cichewicz MA, Dey BK, Layer R, Reon BJ, Gagan JR, Dutta A (2015). MUNC, a Long Noncoding RNA That Facilitates the Function of MyoD in Skeletal Myogenesis. Molecular and Cellular Biology.

[ref-31] Nagel D, Vincendeau M, Eitelhuber AC, Krappmann D (2014). Mechanisms and consequences of constitutive NF-κB activation in B-cell lymphoid malignancies. Oncogene.

[ref-32] Pan J, Liu S, Farkas M, Consugar M, Zack DJ, Kozak I, Fernando Arevalo J, Pierce E, Qian J, Al Kahtani E (2016). Serum molecular signature for proliferative diabetic retinopathy in Saudi patients with type 2 diabetes. Molecular Vision.

[ref-33] Pierzchala M, Hoekman AJW, Urbanski P, Kruijt L, Kristensen L, Young JF, Oksbjerg N, Goluch D, Te Pas MFW (2014). Validation of biomarkers for loin meat quality (M. longissimus) of pigs. Journal of Animal Breeding and Genetics.

[ref-34] Poliseno L (2012). Pseudogenes: newly discovered players in human cancer. Science Signaling.

[ref-35] Polonifi A, Politou M, Kalotychou V, Xiromeritis K, Tsironi M, Berdoukas V, Vaiopoulos G, Aessopos A (2010). Iron metabolism gene expression in human skeletal muscle. Blood Cells, Molecules & Diseases.

[ref-36] Risso D, Ngai J, Speed TP, Dudoit S (2014). Normalization of RNA-seq data using factor analysis of control genes or samples. Nature Biotechnology.

[ref-37] Saeed AI, Sharov V, White J, Li J, Liang W, Bhagabati N, Braisted J, Klapa M, Currier T, Thiagarajan M, Sturn A, Snuffin M, Rezantsev A, Popov D, Ryltsov A, Kostukovich E, Borisovsky I, Liu Z, Vinsavich A, Trush V, Quackenbush J (2003). TM4: a free, open-source system for microarray data management and analysis. BioTechniques.

[ref-38] Seferian AM, Malfatti E, Bosson C, Pelletier L, Taytard J, Forin V, Gidaro T, Gargaun E, Carlier P, Fauré J, Romero NB, Rendu J, Servais L (2016). Mild clinical presentation in KLHL40-related nemaline myopathy (NEM 8). Neuromuscular Disorders.

[ref-39] Shannon P, Markiel A, Ozier O, Baliga NS, Wang JT, Ramage D, Amin N, Schwikowski B, Ideker T (2003). Cytoscape: a software Environment for integrated models of biomolecular interaction networks. Genome Research.

[ref-40] Skurat AV, Dietrich AD, Zhai L, Roach PJ (2002). GNIP, a novel protein that binds and activates glycogenin, the self-glucosylating initiator of glycogen biosynthesis. Journal of Biological Chemistry.

[ref-41] Sun L, Bai M, Xiang L, Zhang G, Ma W, Jiang H (2016a). Comparative transcriptome profiling of longissimus muscle tissues from Qianhua Mutton Merino and Small Tail Han sheep. Scientific Reports.

[ref-42] Sun Y, Wang G, Ji Z, Chao T, Liu Z, Wang X, Liu G, Wu C, Wang J (2016b). Three slow skeletal muscle troponin genes in small-tailed Han sheep (*Ovis aries*): molecular cloning, characterization and expression analysis. Molecular Biology Reports.

[ref-43] Szklarczyk D, Franceschini A, Wyder S, Forslund K, Heller D, Huerta-Cepas J, Simonovic M, Roth A, Santos A, Tsafou KP, Kuhn M, Bork P, Jensen LJ, Von Mering C (2015). STRING v10: Protein–protein interaction networks, integrated over the tree of life. Nucleic Acids Research.

[ref-44] Trapnell C, Williams BA, Pertea G, Mortazavi A, Kwan G, Van Baren MJ, Salzberg SL, Wold BJ, Pachter L (2010). Transcript assembly and quantification by RNA-Seq reveals unannotated transcripts and isoform switching during cell differentiation. Nature Biotechnology.

[ref-45] Tu YR (1989). The sheep and goat breeds in China.

[ref-46] Wang L, Zhao Y, Bao X, Zhu X, Kwok YKY, Sun K, Chen X, Huang Y, Jauch R, Esteban MA, Sun H, Wang H (2015). LncRNA Dum interacts with Dnmts to regulate Dppa2 expression during myogenic differentiation and muscle regeneration. Cell Research.

[ref-47] Winter JMD, Joureau B, Lee EJ, Kiss B, Yuen M, Gupta VA, Pappas CT, Gregorio CC, Stienen GJM, Edvardson S, Wallgren-Pettersson C, Lehtokari VL, Pelin K, Malfatti E, Romero NB, Engelen BGV, Voermans NC, Donkervoort S, Bönnemann CG, Clarke NF, Beggs AH, Granzier H, Ottenheijm CAC (2016). Mutation-specific effects on thin filament length in thin filament myopathy. Annals of Neurology.

[ref-48] Xu J, Hwang JCY, Lees HA, Wohlgemuth SE, Knutson MD, Judge AR, Dupont-Versteegden EE, Marzetti E, Leeuwenburgh C (2012). Long-term perturbation of muscle iron homeostasis following hindlimb suspension in old rats is associated with high levels of oxidative stress and impaired recovery from atrophy. Experimental Gerontology.

[ref-49] Xu Q, Chen Y, Zhang Y, Tong YY, Huang ZY, Zhao WM, Duan XJ, Li X, Chang G Bin, Chen GH (2014). Molecular cloning and expression analysis of ferritin, heavy polypeptide 1 gene from duck (Anas platyrhynchos). Molecular Biology Reports.

[ref-50] Xu X, Ji S, Li W, Yi B, Li H, Zhang H, Ma W (2017). LncRNA H19 promotes the differentiation of bovine skeletal muscle satellite cells by suppressing Sirt1/FoxO1. Cellular and Molecular Biology Letters.

[ref-51] Yue Y, Guo T, Yuan C, Liu J, Guo J, Feng R, Niu C, Sun X, Yang B (2016). Integrated analysis of the roles of long noncoding RNA and coding RNA expression in sheep (*Ovis aries*) skin during initiation of secondary hair follicle. PLOS ONE.

[ref-52] Zhan S, Dong Y, Zhao W, Guo J, Zhong T, Wang L, Li L, Zhang H (2016). Genome-wide identification and characterization of long non-coding RNAs in developmental skeletal muscle of fetal goat. BMC Genomics.

[ref-53] Zhang C, Wang G, Wang J, Ji Z, Dong F, Chao T (2014). Analysis of differential gene expression and novel transcript units of ovine muscle transcriptomes. PLOS ONE.

[ref-54] Zhu J, Sanborn JZ, Diekhans M, Lowe CB, Pringle TH, Haussler D (2007). Comparative genomics search for losses of long-established genes on the human lineage. PLOS Computational Biology.

